# A Change in Vaccine Efficacy and Duration of Protection Explains Recent Rises in Pertussis Incidence in the United States

**DOI:** 10.1371/journal.pcbi.1004138

**Published:** 2015-04-23

**Authors:** Manoj Gambhir, Thomas A. Clark, Simon Cauchemez, Sara Y. Tartof, David L. Swerdlow, Neil M. Ferguson

**Affiliations:** 1 Department of Epidemiology and Preventive Medicine, Monash University, Melbourne, Australia; 2 Modeling Unit, National Center for Immunization and Respiratory Diseases (NCIRD), Centers for Disease Control and Prevention (CDC), Atlanta, Georgia, United States of America; 3 IHRC, Inc., Atlanta, Georgia, United States of America; 4 Meningitis and Vaccine Preventable Diseases Branch, Division of Bacterial Diseases, NCIRD, CDC, Atlanta, Georgia, United States of America; 5 Medical Research Council Centre for Outbreak Analysis and Modelling, Imperial College London, London, United Kingdom; 6 Mathematical Modelling of Infectious Diseases Unit, Institut Pasteur, Paris, France; 7 Kaiser Permanente Southern California, Kaiser Permanente Research, Department of Research & Evaluation, Pasadena, California, United States of America; 8 Office of Science and Integrative Programs, NCIRD, CDC, Atlanta, Georgia, United States of America; Pennsylvania State University, UNITED STATES

## Abstract

Over the past ten years the incidence of pertussis in the United States (U.S.) has risen steadily, with 2012 seeing the highest case number since 1955. There has also been a shift over the same time period in the age group reporting the largest number of cases (aside from infants), from adolescents to 7–11 year olds. We use epidemiological modelling and a large case incidence dataset to explain the upsurge. We investigate several hypotheses for the upsurge in pertussis cases by fitting a suite of dynamic epidemiological models to incidence data from the National Notifiable Disease Surveillance System (NNDSS) between 1990–2009, as well as incidence data from a variety of sources from 1950–1989. We find that: the best-fitting model is one in which vaccine efficacy and duration of protection of the acellular pertussis (aP) vaccine is lower than that of the whole-cell (wP) vaccine, (efficacy of the first three doses 80% [95% CI: 78%, 82%] versus 90% [95% CI: 87%, 94%]), increasing the rate at which disease is reported to NNDSS is not sufficient to explain the upsurge and 3) 2010–2012 disease incidence is predicted well. In this study, we use all available U.S. surveillance data to: 1) fit a set of mathematical models and determine which best explains these data and 2) determine the epidemiological and vaccine-related parameter values of this model. We find evidence of a difference in efficacy and duration of protection between the two vaccine types, wP and aP (aP efficacy and duration lower than wP). Future refinement of the model presented here will allow for an exploration of alternative vaccination strategies such as different age-spacings, further booster doses, and cocooning.

## Author Summary

Over the past ten years the incidence of pertussis in the United States (U.S.) has risen steadily, with 2012 seeing the highest case number since 1955. There has also been a shift over the same time period in the age group reporting the largest number of cases (aside from infants), from adolescents to 7–11 year olds. We investigate several hypotheses for the upsurge in pertussis cases by fitting a suite of epidemiological models to incidence data from the National Notifiable Disease Surveillance System (NNDSS) between 1990–2009. We find that: 1) the best-fitting model is one in which the vaccine efficacy and duration of protection of the acellular pertussis vaccine is lower than that of the whole-cell vaccine, 2) increasing the rate at which disease is reported to NNDSS is not sufficient to explain the upsurge and 3) 2010–2012 disease incidence is predicted well. These results demonstrate that the resurgence in pertussis in the U.S. can be explained by past changes in vaccination policy. However, our findings suggest that the efficacy of the currently-used acellular vaccine is not much lower than that of the whole-cell vaccine, and booster doses may be sufficient to curtail epidemics while vaccine research continues.

## Introduction

In 2012, the United States experienced the highest number of reported pertussis cases since 1955, shortly after the introduction of vaccine in the 1940s [1–3]. This upsurge occurred in the context of steadily rising reported disease from the early-1980s [4], despite the maintenance of high vaccination coverage levels (>90%) [5]. Pertussis remains a major cause of childhood mortality world-wide, responsible for 195,000 deaths in 2008 [6]. A vaccine for the disease was developed in 1942 in the U.S., and was included and administered as a killed whole-cell component of the diphtheria/tetanus/pertussis (DTP) combination vaccine.

**Fig 1:**
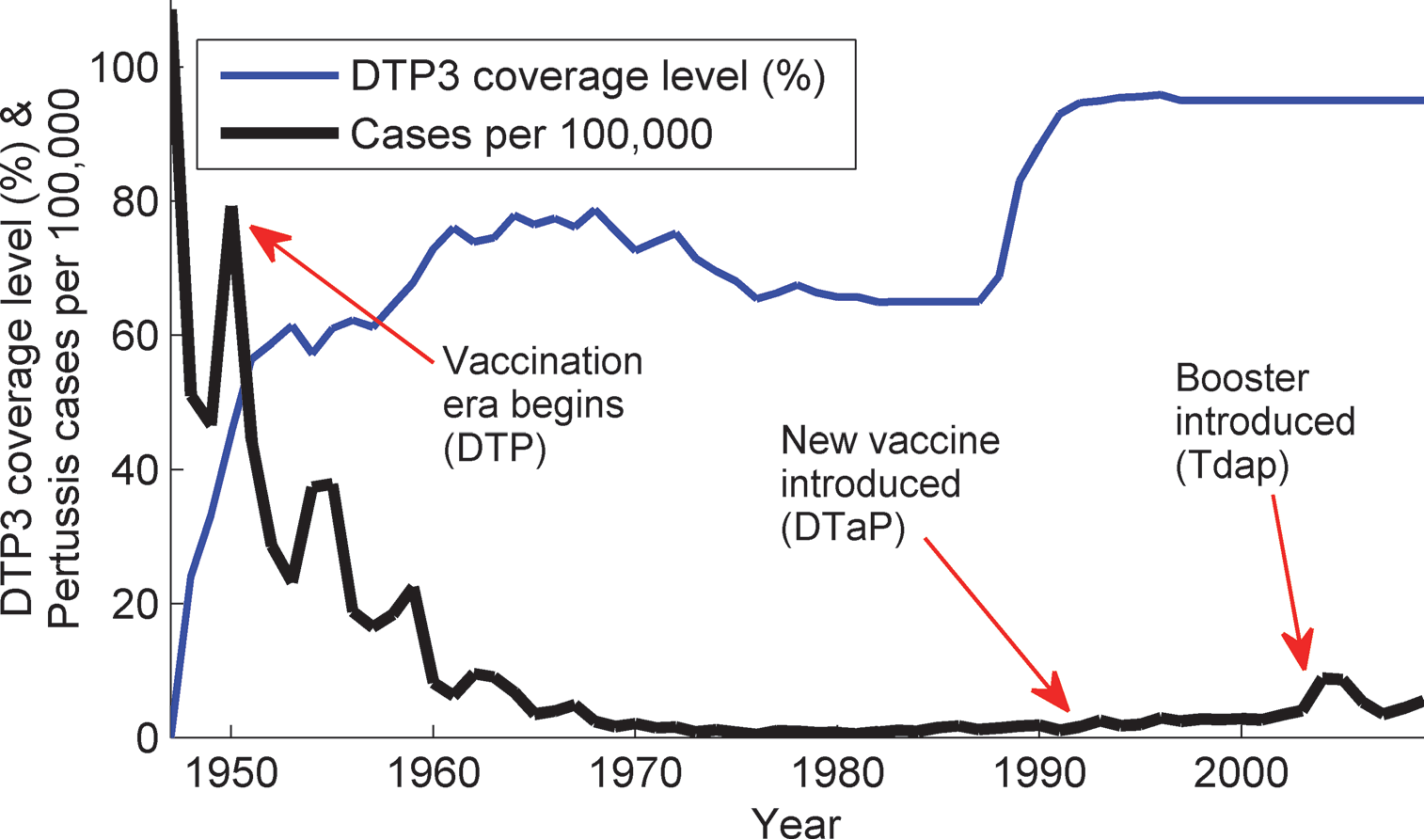
Incidence of disease and vaccination coverage in the United States. The time varying incidence of disease in the US, summed over all ages, between 1950 and 2009, annotated for significant events relating to vaccination policy. The blue solid line illustrates the DTP3 (first three DTP doses) vaccine coverage level over time.

Following introduction of vaccination, the reported disease incidence in the U.S. declined from 150 cases per 100,000 per annum prior to 1940, to the point of near elimination in the mid-1970s (0.5 reported cases per 100,000)[4] (Fig 1). Due to the possible reactogenicity of the whole-cell pertussis component of the DTP vaccine, acellular pertussis (aP) vaccine was developed as a replacement in 1991 (DTP became DTaP). This new vaccine was administered to children in 1992 [7,8] and then phased into the infant immunization schedule beginning in 1997 [9,10], following approval by the U.S. Food and Drug Administration (FDA) and recommendations by the U.S. Advisory Committee on Immunization Practices (ACIP).

Over the past 30 years, reported pertussis incidence in the U.S. has been steadily increasing (Fig 1). Even accounting for this steady upward trend, which some have attributed to improved surveillance and diagnostics [11,12], case numbers during 2004–2012 were notably high and many state public health departments reported outbreaks during these years. During the two most recent of these outbreaks, the worst-hit states were California (in 2010) [2,3] and Washington (in 2012) [13] with case counts unmatched since the mid-1940s. While the 2004–2005 outbreak was characterized by an increase in the number of cases among adolescents (10–20 year olds), as well as a less-pronounced increase across all ages, the most recent outbreaks have seen many more cases among 7–10 year olds and young adolescents (11–13 year olds) along with an overall increase in case numbers [2].

A number of explanations have been put forward for the increase in disease that has been observed, particularly for the outbreaks of the past 10 years [11,14–19]. These include: 1) The evolution of the circulating bacteria away from the targets of vaccine antigens; 2) A decline in vaccine coverage levels; 3) a change in vaccine efficacy (VE), and/or duration of protection due to the shift to the acellular vaccine; or a specific lot of manufactured vaccine with lower efficacy; 4) The decline of natural boosting, through reduced exposure to naturally circulating pertussis bacteria, as a result of years of successful mass-vaccination; 5) An increase in disease reporting rates (e.g. due to improved diagnostics and surveillance) [20,21].

We examine these possible explanations using a population dynamic model representing infection with *Bordetella pertussis* in an age-structured population. We fit the output of this model to 20 years of age-stratified incidence data from the National Notifiable Diseases Surveillance System (NNDSS). The model includes both vaccine and naturally-induced immunity (with both of these sources of protection waning over time), the current vaccination schedule administered to the population, an estimate of the underreporting of disease, and a decreased infectiousness of those who have experienced greater than one infection.

## Results

Eight different (nested) models were fitted to the NNDSS incidence data (Table 1). The most parsimonious (best fitting) model, Model 8, has a difference in efficacy and duration of protection between the whole-cell and acellular vaccines, and a difference between the duration of protection of whole-cell vaccine and natural infection (Table 1). Model 5, in which the duration of protection for whole-cell vaccine and natural infection is the same, has a slightly lower DIC value, but is very close to Model 8, and these two models are almost interchangeable in terms of their parameter values. Further details of the candidate models are provided in the Supporting Information.

**Table 1:** Descriptions of the nested models that were fitted to the NNDSS incidence data.

Model	Description	DIC
1	Protection duration of whole cell vaccine same as natural infection; acellular vaccine same as whole-cell	-9720
2	Protection duration of whole cell vaccine same as natural infection; different efficacy for acellular vaccine	-9570
3	Protection duration of whole cell vaccine same as natural infection; different protection duration for acellular vaccine;	-9250
4	Protection duration of whole cell vaccine different from natural infection; acellular vaccine same as whole-cell	-9800
5	Protection duration of whole cell vaccine same as natural infection; protection duration and efficacy different for acellular vaccine	-8422
6	Whole cell vaccine protection duration different from natural infection; different efficacy for acellular vaccine	-9183
7	Whole cell vaccine protection duration different from natural infection; different protection duration for acellular vaccine	-9230
8	Whole cell vaccine protection duration different from natural infection; protection duration and efficacy different for acellular vaccine	-8417

The mean posterior values of the Deviance Information Criterion (DIC) of the models are given in the rightmost column.

**Fig 2:**
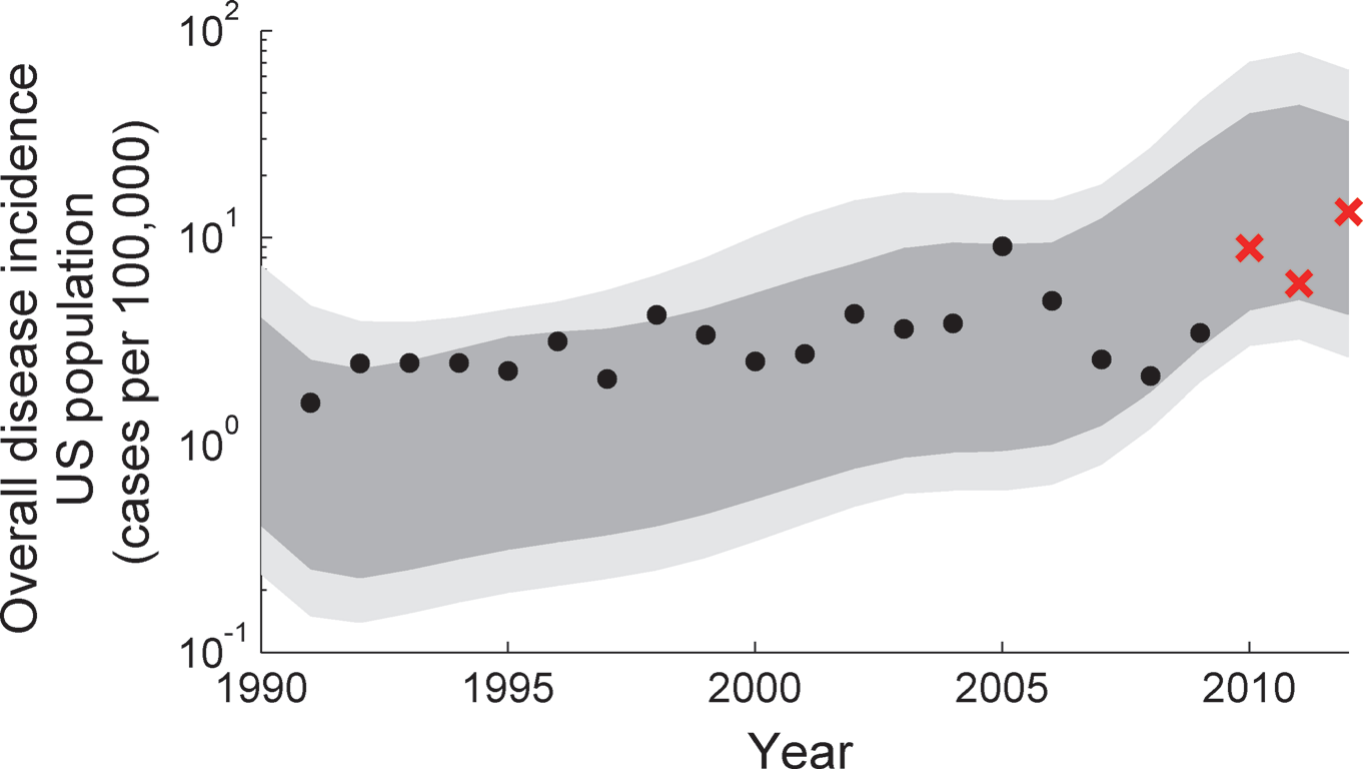
Incidence of disease in the United States, compared with modeled values. The time varying incidence of disease cases in the model and the US data (log scale) after 1990. The black dots are US disease incidence data, and the shaded regions represent the credible intervals (50% and 95%) obtained through model parameter estimation of model 8. The model has been run beyond the time over which it was trained to illustrate its continued correspondence with the 2010–2012 data (red crosses).

The model reproduces the trends in the NNDSS incidence data up to 2009; running the model forward over another three years shows a continued correspondence with observed incidence trends (Fig 2). A large proportion of forward simulations (>50%) show an upsurge in incidence around 1990, a phenomenon that is not recorded in the data. However, the mean of the model-predicted incidence remains much closer to the data, albeit with a large range of uncertainty.

**Fig 3:**
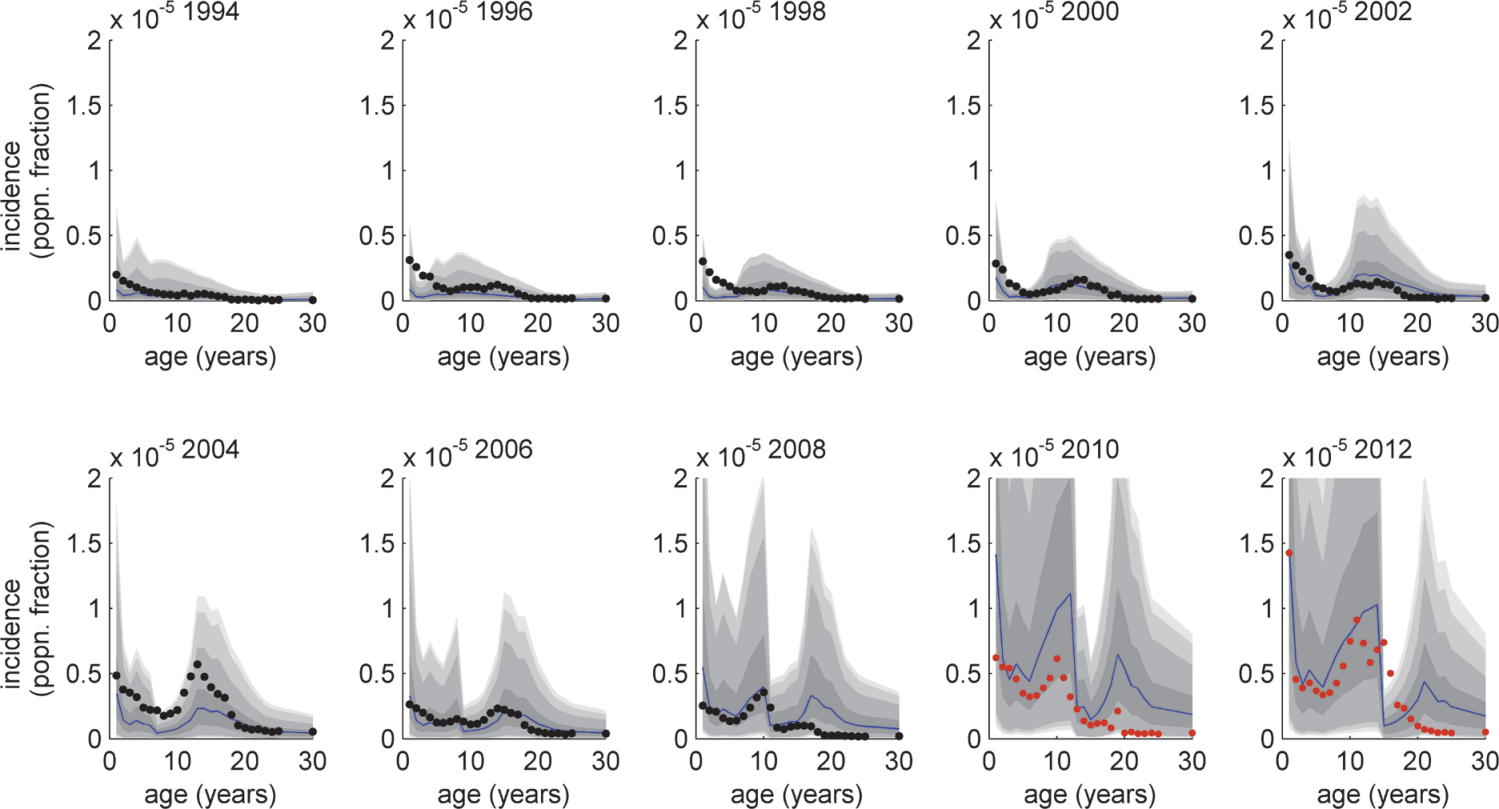
Cross-sectional incidence of disease over age of population. Annual age-dependent (to age 30 years) incidence of pertussis disease cases for the years 1994–2012 in 2 year intervals. Gray shaded envelopes indicate 50%, 90%, 95%, and 99% credible intervals from the model parameter and uncertainty estimation. Black points (1994–2008) indicate disease incidence data collected by the NNDSS. The red crosses on the 2010 and 2012 plots also represent NNDSS incidence data; however, the model was not fitted to these data and so model outputs represent out-of-sample predicted age-dependent incidence curves.

Fig 3 illustrates the age distribution of incident cases every two years from 1994–2012, for Model 8. Several features of the NNDSS data are reproduced by the model: 1) an overall rise in disease cases throughout the observed period; 2) an adolescent peak in the incidence curve for most of the years post-1995; 3) a gradual rise in cases among 5–10 year olds from 2006, forming a peak which is comparable in size with the adolescent peak by 2008.

In 2002, the dominant peak in incidence is among adolescents but another smaller peak emerges among infants. This smaller peak moves forward one year at a time, growing in magnitude until 2006 when the two peaks are of similar size. By 2008, the cohort of individuals immediately following the shift in vaccine efficacy have advanced to become 10 years of age and they constitute the 'wavefront' of a swell of new disease. The two plots for 2010 and 2012 are included to illustrate that the model continues to correspond well with the age-distribution of disease beyond the time period for which it was fitted (i.e. beyond 2009). Note that the correspondence between these ‘predicted’ distributions of disease with age was not quantitatively determined, due to the complexity of the total likelihood used for model-fitting, but these distributions continued to capture the main features of the disease profile, namely: a) a rising overall incidence; b) the highest peak of disease among infants; c) a growing adolescent peak moving up in age with each advancing year.

The best-fitting model incorporates a drop in vaccine efficacy and a rise in the vaccine-protection waning rate from the whole cell to acellular vaccine. Both the change in the per dose efficacy and the duration of protection change appreciably between vaccine types. Table 2 shows the estimated mean and 95% confidence intervals (CI) of the efficacies of the first three doses, considered together, and the fourth and fifth doses (the probability of fully protective seroconversion).

**Table 2:** Parameter estimates for the best-fitting model, Model 8 (models outlined in Table 1).

Parameter description	Value
**Vaccine efficacies & waning**	
***Whole-cell***	
Vaccine efficacy of 1^st^ 3 doses/4^th^/5^th^ dose	90% [87%, 94%]
Rate of loss of whole-cell vaccine immunity	3x10^-5^yr^-1^ [2x10^-6^, 2x10^-4^] i.e. essentially lifelong
***Acellular***	
Vaccine efficacy of 1^st^ 3 doses/4^th^/5^th^ dose	80% [78%,82%]
Rate of loss of acellular vaccine immunity	0.018yr^-1^ [0.015, 0.020] i.e. average of approx. 50 yrs protection
***Tdap***	
Vaccine efficacy	As acellular
**Epidemiological Parameters**	
Basic reproduction number, *R*_0_	11.0 [9.9, 11.5]
Rate of loss of natural immunity	3x10^-5^yr^-1^ [2x10^-6^, 2x10^-4^] i.e. essentially lifelong (as for whole-cell)
Relative susceptibility of individuals to subsequent infection (with reference to naïve individuals)	32% [29%, 35%]
Relative infectiousness of individuals with subsequent infection (with reference to primary-infected individuals)	17% [14%, 23%]
Year of reporting rate change	None
Mean reporting rate prior to change	6.0% [0.1%, 22%]
Mean reporting rate after change	n/a

Estimates of the epidemiological parameters are shown in Table 2. The basic reproduction number, *R*_0_, was estimated to be 11 [95% CI: 9.9, 11.5], slightly lower than some previous estimates [18]. Secondary and subsequent infections (those which are experienced by individuals who retain some level of immune protection, whether due to vaccination or infection-induced immunity) are found to be 32% [95% CI: 29%, 35%] as infectious as primary infections and these individuals are found to be 17% [95% CI: 14%, 23%] as susceptible to infection as infection-naïve individuals.

The disease reporting rate is included in the model as a fraction of the infected cases experiencing primary infection who will be symptomatic and captured by NNDSS surveillance. We find that the best-fitting model does not require a change in the reporting rate to reproduce the major trends in the data. The reporting rate is estimated at 6% [95% CI: 0.1%, 22%] with the wide credible intervals suggesting that a gradual change in reporting over time may not be precluded, even if a step change is not supported. A gradual change in reporting is consistent with the uptake of more sensitive diagnostic methods as well as greater awareness of pertussis in recent years.

**Fig 4:**
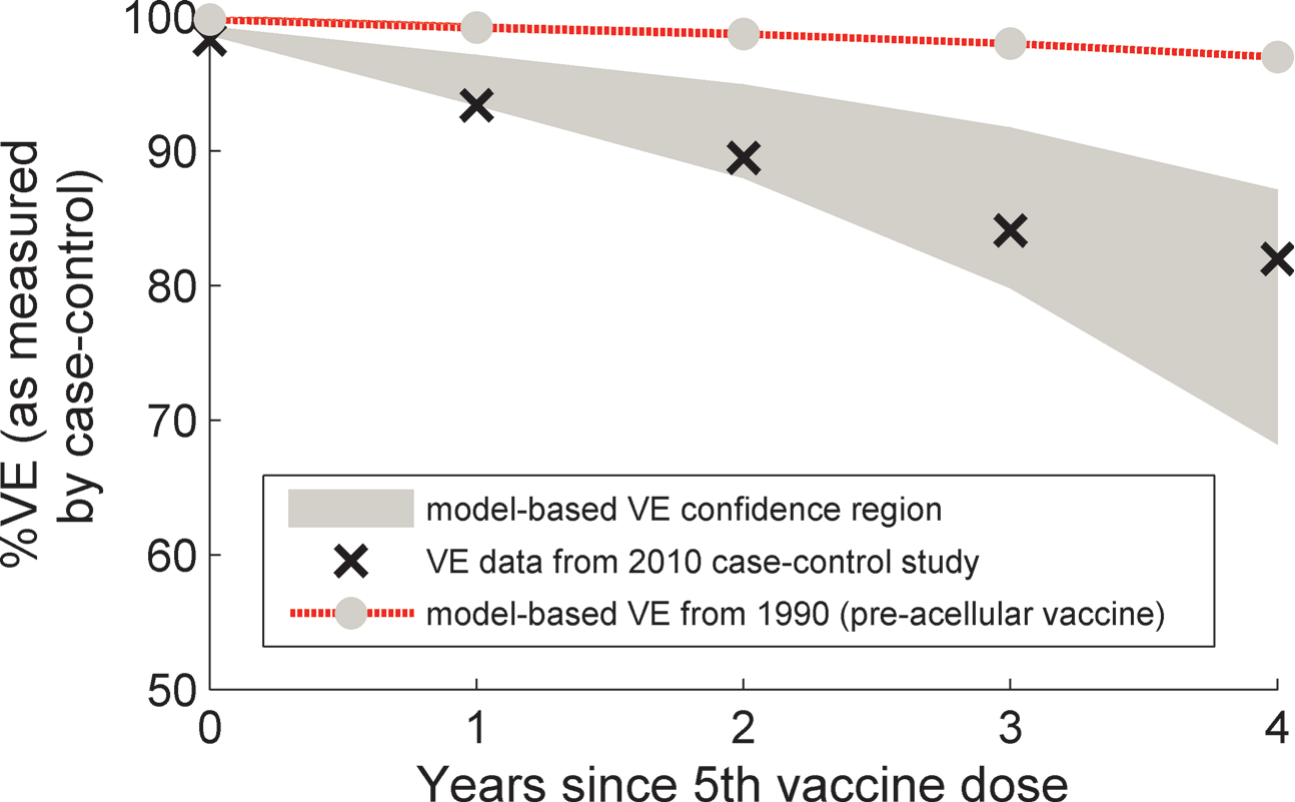
Case-control study results compared with modeled values. Vaccine Effectiveness (VE) as measured in the case-control study of Misegades et al [22] from 2010 in California (black crosses) compared with VE values generated by simulations of the case-control study using the model fitted to the incidence and these VE data (gray region). The gray shaded region represents the 95% credible interval of the model outputs. The dotted curve lying above the 2010 data and simulations was calculated by simulating a 1990 case-control study, and shows significantly slower waning of the VE value.

Fig 4 shows the results obtained by simulating the case-control study conducted in 2010 by Misegades et al [22]. We demonstrate that, in successive ages beyond when the final vaccine dose is administered, there is a decline in the 95% credibility envelope of the model-based VE estimates (gray-shaded region): the bottom of this envelope declines more rapidly than measured for the California study. The curve lying above the shaded region illustrates the results of a hypothetical VE study if it had been conducted in 1990, the pre-acellular era. It can be seen that VE declines more slowly in this case.

## Discussion

We have shown that relatively small differences in the per-dose vaccine efficacy and duration of protection between acellular and whole-cell vaccines is sufficient to explain the recent upsurge in pertussis in the United States. These differences caused a shift in the age distribution of pertussis disease incidence: the adolescent peak in years prior to 2006 shifted to a younger age group (5–10 year olds) post-2006. This explanation for pertussis disease dynamics is in broad agreement with case-control and retrospective cohort studies showing that vaccine-induced immunity wanes faster than previously thought [22–24].

**Fig 5:**
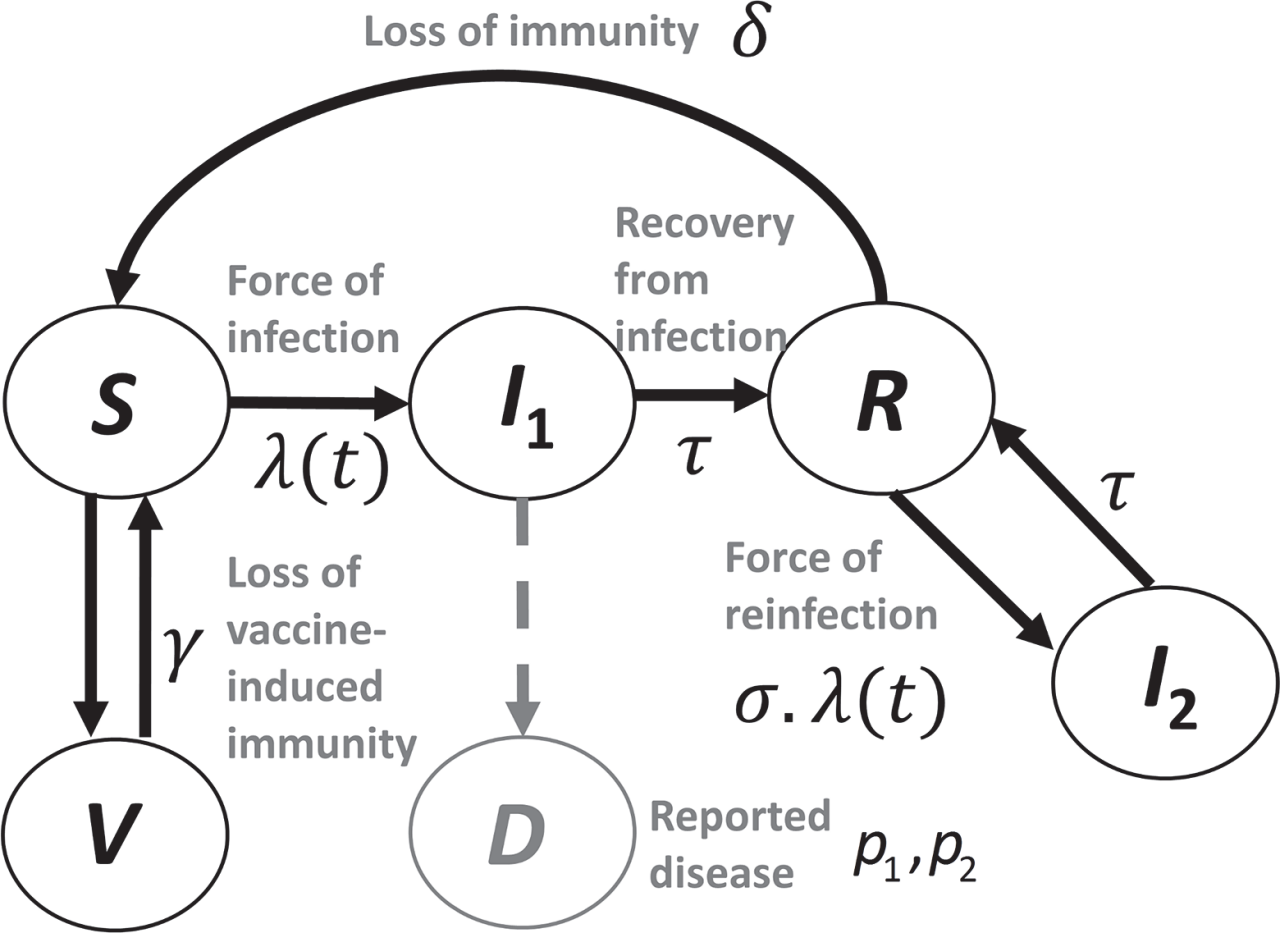
Epidemiological model diagram. The compartmental model for pertussis infection and disease used, modified from the basic model of Aguas *et al* [44]. There are two infected compartments for primary (*I*_1_) and secondary (or higher) infection (*I*_2_). We assume that surveillance systems only capture those who are experiencing primary infection. Once an individual has recovered from primary or secondary infection, induced immunity may wane, whereupon they will re-enter the susceptible state. Rates of flow between compartments are defined in Table 3.

The average duration of protection we estimate for acellular vaccine is shorter than the essentially lifelong protection we estimate for both whole-cell vaccination and natural infection; however our estimate of the acellular protection duration is around 50 years, which is longer than might be expected from published VE studies [22,23]. Since our calculated waning rate is an average over the whole population, this means some individuals will re-enter the susceptible pool more quickly than others. By simulating a prospective VE study, we find that even a small increased flow into the susceptible pool is sufficient to result in a strong signal of declining VE following the final vaccine dose (see Figs 4 and 5 and [22]). The efficacy values implied here for the full acellular vaccine regimen (i.e. 5 doses) are consistent with those estimated in the acellular pertussis vaccine (APERT) trial [25], lending further weight to the correspondence of our model with the broad range of data. Furthermore, VE values for individuals with mixed acellular and whole-cell vaccine histories are consistent with a recent Australian report of disease rates and linked vaccination histories [26].

While our analysis does not preclude the possibility of a rise over time in the rate of reporting (or detection) of cases of disease, such a rise is not necessary to explain recent patterns in pertussis incidence. Our estimate of case reporting rates (approximately 6%) is in line with a recent multi-country analysis [20], but has wide 95% credible intervals. Hence, progressive changes in disease reporting, such as those expected by more sensitive diagnostic testing (e.g. PCR) or a greater awareness of pertussis among patients and physicians, is within the scope of our model. Further work with this model could investigate published reporting trends more closely [11,20,21,27,28].

Our analysis sheds light on other, previously uncertain, aspects of pertussis epidemiology. First, we estimate *R*_0_ to be in the range of 9–12 which is closer to the values found by previous mathematical models [29,30] than the often-quoted range of 12–17 [31]. Such values do not rule out elimination at high enough vaccination coverage levels. Second, previous recipients of vaccine whose protection has waned are estimated to be 32% as susceptible to infection as infection-naïve individuals and, if infected, these individuals are estimated to be 17% as infectious as primary infections in immunologically naïve individuals. These numbers suggest that adults and adolescents may be an important reservoir of infection. Third, the most parsimonious model with a good fit to the data suggests that the duration of immunity generated by the whole-cell vaccine is nearly equivalent to that induced by natural infection.

Our explanation for the upsurge and the age-shift in disease incidence differs from other recent model-based studies [14,15]. We find a 'hypersensitive' boosting model [14] to be too unstable to mimic the steady post-1970s rise in disease; the incidence of disease for that model shows a large amplitude oscillatory behaviour. Population age-dependent contact patterns alone also appear to be insufficient to capture recent changes in U.S. incidence of disease [15]. Declines in vaccination coverage and the evolution of the circulating bacteria would lead to rising disease but not the changing age-distribution, although the recent discovery of pertactin-negative (or other genetic) variants may be playing a role in the vaccine efficacy decline we have found [32,33]. Changes in age-dependent disease reporting could mimic observed incidence patterns, of course, but such models would lack the simplicity of ours.

Future refinement of the model presented here will allow for an exploration of alternative vaccination age-spacings for the five-dose childhood schedule, investigation into the effects of further booster doses (for example, decennially to adults) and cocooning vaccination strategies designed to protect infants who are most vulnerable to severe disease [22]. Further data collection will help to narrow uncertainties in the model, for example, the range of *R*_0_ and, the infectiousness of those who have been previously infected, both relevant to control strategies. Alternative model structures capturing the development and waning of immunity and boosting of immunity may be contributing to discrepancies between the outputs of our best-fitting model and the available data, such as an overestimate of the incidence peak in adolescents after 2008 (Fig 3), and signs of a model underestimate of the overall incidence (Fig 2). Future work should attempt to incorporate the full range of possibilities for modeling boosting and immunity e.g. using the proposed model of Lavine et al. [14], as well as mixing matrix data that are as locally-specific as possible, following the work of Rohani et al. and Riolo et al. [15,34]. Clearly, the use of a mixing matrix from Great Britain is not ideal for the U.S. but was all that was available at the time of writing. In addition, fitting our model to data from the U.S. aggregates the incidence from a large number of more spatially localized epidemics, which may not be completely synchronized; this means that, while our model is convincing, it tends to describe phenomena such as the ‘cohort effect’ and peaks in incidence more sharply than the data. Future work should focus on modeling local outbreaks and endemic pertussis with high spatial resolution, following Rohani et al. and Choisy et al. [35,36]. More intricate modeling frameworks, such as individual-based modeling, are also promising directions for future work, given their ability to represent human behavioural dynamics, which could be a major local contributor to vaccine uptake or refusal [37].

A further important step for this study is the analysis of pertussis incidence data—along with demography and vaccination histories—from around the world. Jackson et al. recently reviewed such data and showed that global pertussis epidemiology is complicated and by no means follows the same patterns and explanations as the U.S. [38]. However, the authors of the review do note that the issue of the switch from the wP to the aP vaccine may be sufficiently specific to allow investigation of its impact upon global trends.

Transmission models with a realistic representation of the natural history of infection and immunity, and population contact processes, fitted to data with statistically rigorous methods, are useful tools to quickly investigate increases in disease incidence, whether they occur within the U.S. or globally [39–41]. Pertussis epidemiology is complex and disease incidence is non-linearly related to vaccine coverage. With doubt around the efficacy and duration of protection of the acellular vaccine, modeling is an essential tool to help us better understand the changing epidemiology of pertussis.

## Methods

We constructed a compartmental mathematical model of the natural history and population transmission of *B*. *pertussis* infection in the United States, in which the population transitions between three states of infection: susceptible, infected, and recovered from a prior infection. Infected individuals can be either primary-infected or infected more than once (see Fig 5, Table 3 and ‘Model structure details’ below). The model also accounts for the annual aging of the population: the time-dependent birthrates and deathrates are used for the U.S. (obtained from [42]). The birth and deathrates prior to the analysis period of the study were fixed at the earliest values available, namely those of 1950. Mixing between age-groups is incorporated according to a European diary-based contact study (used in the absence of a suitable U.S. alternative [43]).

**Table 3:** State variables (population compartments) and model parameters for the model investigated.

Symbol	Description	Value
***Population compartments (state variables)***		
*S*	Susceptible	
*I*_1_	Primary infection	
*I*_2_	Secondary (or greater) infection	
*R*	Recovered from infection (whether primary or secondary)	
*V*	Vaccinated	
*D*	Reported cases of pertussis	
***Model parameters***		
*λ*(*t*)	Force of infection at time *t* (this subsumes the model parameter β (known as the transmission parameter) which is estimated by the model-fitting procedure)	Varies in value depending upon number of individuals infected (Per capita per year)
*Τ*	Rate of recovery from primary or secondary infection	24 per year (i.e. duration of infection is approx. 1/24 years = 16 days)
*Δ*	Rate of loss of immunity following natural infection	Estimated here (see Table 2)
*Γ*	Rate of loss of immunity following vaccination	Estimated here (see Table 2)
*Σ*	Relative rate of acquisition of secondary to primary infection	Estimated here
*Η*	Relative infectiousness of secondary infections	Estimated here
*p1*, *p2*	Rate at which infected individuals report disease	Estimated here

Vaccination is included as a process by which a proportion of the susceptible population at a particular age is transferred to either the recovered compartment or a vaccinated compartment (i.e. *all-or-nothing* vaccination protection for each dose). In both of these compartments, individuals have developed a protective immune response which decreases the probability of infection but eventually wanes. This vaccinated proportion may be thought of as *coverage* x *vaccine efficacy* and is equal to the protected proportion of the population with each dose. Annual vaccination coverage levels were obtained from the National Immunization Survey and its predecessor surveys [45].

The U.S. pertussis vaccine schedule recommends three doses to infants—at two, four and six months and two further doses: one at 18 months and the fifth and final dose between four and six years of age. We model the first three doses as a *single* removal of susceptible infants six months following birth (as an approximation of the building immunity due to the first three doses), the 18 month dose as *two* separate removals, each with one half of the overall coverage, at one and two years of age and, similarly, the final dose as *two* separate removals at four and five years of age. The 18 month and four to six year doses are represented as double doses to capture a fairly wide range of times at which vaccine is administered to children across the whole population. The whole-cell vaccine is first applied to the model population in the simulation year 1947 and a switch is made to the acellular vaccine for the final two doses in 1992 and then to the entire schedule in 1998. A Tdap booster vaccine is introduced to those aged 12 years in the model, in 2005, with coverage levels given by recent surveys [46–51].

Disease is reported at a rate, *p*, the proportion of cases captured by NNDSS. This rate was permitted to change once in the time period modelled, to partially account for (i) a possible change in the sensitivity with which cases are found over time, and (ii) increased reporting associated with greater awareness of pertussis among patients and physicians. We also allow for some level of spatial and temporal heterogeneity in the reporting rate by modelling *p* as drawn from a Beta probability distribution (see Statistical details); this allows for a single average value of the reporting rate to be estimated for each of the two time periods, but for the uncertainty in this value to be quantified. Only those in the primary-infected compartment (*I*_1_, Fig 5) were counted as disease cases; this is a simplification, but one that is justified by recent studies which show that secondary-infected or vaccinated non-human primates had more limited disease when reinfected [52].

Models were fitted in a Bayesian framework to annual incidence data, summed over the whole U.S. population (in cases per 100,000 persons), from 1950–1989, and to age-stratified incidence data obtained from NNDSS for the years 1990 to 2009. The fitting was performed using Markov Chain Monte Carlo (MCMC) methods, which find an ensemble of model parameters that fit the data and allow a credible interval for each of these parameters to be determined. The chains were run to 100,000 steps during equilibration and 50,000 to sample the posterior parameter distributions. Sensitivity of the parameter estimates was explored by performing MCMC separately using different subsets of the data.

Several candidate models were constructed, each with the basic structure given in Fig 5 but including or excluding specific components, and their parameters were fitted to the data (see Table 1). These model components included possible changes in (i) duration of protection between whole-cell vaccination and natural infection; (ii) vaccine efficacy and (iii) duration of protection between the whole-cell and acellular vaccine. The candidate models were compared to determine the simplest and most biologically compelling explanation of the observed variations using the Deviance Information Criterion (DIC), a measure that accounts for both goodness-of-fit as well as model complexity [53] (the higher the value, the better the fit to the data).

Misegades et al [22] performed a case-control study in California in 2010 to determine the vaccine effectiveness (VE)(of the full course of 5 doses of DTaP for each of 5 years following the delivery of the final dose. In order to further constrain our parameter selections and values, we used our models to simulate this study and fitted the resulting VE values to those found by Misegades at each of the corresponding times. Misegades found that the VE decreased from 98% to 82% in 4 years following the final of five doses. As well as measuring our model-based VE over the same period, we used the same method to estimate VE during a period when the course of doses consisted entirely of the whole-cell vaccine; we selected 1990 for this purpose.

### Model structure details

The mathematical model we constructed has an age-structured susceptible-infected-recovered (*S*_*i*_, *I*_1*i*_, *R*_*i*_) structure (the subscript *i*, corresponds to one of 35 age-groups), but with the addition of a second infected compartment (*I*_2*i*_) to account for those who have been previously infected. The mixing matrix *β*(*i*, *j*) represents the product of the contact rate (obtained from the ‘POLYMOD’ diary study of contact patterns in Great Britain (GB) [43]) and the transmission probability per contact between individuals in age-groups *i* and *j*. Individuals in state *I*_2*i*_ have an infectiousness value of *η* compared with those in state *I*_1*i*_, and the relative susceptibility to infection of those in *R*_*i*_ (the ‘recovered’ state) compared with those completely susceptible. Vaccination occurs with the DTP vaccine with whole-cell pertussis component by shifting individuals into compartment *V*_1*i*_ and into *V*_2*i*_ for the DTaP and Tdap vaccines (the main text simplifies this flow into a single compartment *V*_*i*_ so as not to clutter the exposition). The equations corresponding to the model flow diagram (Fig 5, Table 3) are given below:

$$\begin{array}{c} \frac{dS_{i}}{dt}=-S_{i}.\left(\lambda_{i}+\mu_{i}\right)+\alpha .R_{i}\\ \frac{dI_{1i}}{dt}=S_{i}.\lambda_{i}-(\tau +\mu_{i}).I_{1i}\\ \frac{dI_{2i}}{dt}=\sigma \lambda_{i}.R_{i}-\left(\tau +\mu_{i}\right).I_{2i}\\ \frac{dR_{i}}{dt}=\tau .(I_{1i}+I_{2i})-\left(\delta +\sigma \lambda_{i}+\mu_{i}\right).R_{i}\\ \frac{dV_{1i}}{dt}=-\left(\gamma +\mu_{i}\right).V_{1i}\\ \frac{dV_{2i}}{dt}=-\left(\gamma +\mu_{i}\right).V_{2i}\\ \text{where the force of infection},\;\lambda_{i}=\sum_{j=1}^{35}\beta \left(i,j\right).\left(\frac{I_{1j}}{N_{j}}+\eta .\frac{I_{2j}}{N_{j}}\right) \end{array}$$

### Statistical details

Pertussis case count data from NNDSS were aggregated into annual counts for each age group so that *y*_*i*_ (*t*) is the number of pertussis disease cases for age group *i* in year *t*. Our mathematical model outputs were also aggregated into annual counts for each age group *i*, so that *x*_*i*_ (*t*) was the model-derived case count for age group *i*, during year *t*. These model-derived case counts are functions of the model structure and parameters, so that they might be better expressed as *x*_*i*_ (*t*|***θ***, *M*), where ***θ*** represents the parameter vector for model *M*.

Since the pertussis cases counted by NNDSS may be a proportion of the true number (and the output of the natural history epidemiological model is designed to simulate the true number) we can say that the observed number of cases follows an observation model so that annual counts of pertussis cases (*obs*_*i*_ (*t*)) are governed by a binomial distribution:

$$obs_{i}\left(t\right)=Binomial\left(x_{i}\left(t|\theta ,M\right),\left(t\right),p_{i}\left(t\right)\right)$$

The proportion of individuals with disease who report it (*p*_*i*_ (*t*)) may vary over time in a number of ways (e.g. linear/nonlinear increase or decrease) but here we account for this temporal uncertainty in the simplest way, by allowing the value of *p*_*i*_ (*t*) to change once during the course of a simulation run (i.e. it has one value prior to a time *T* and a different value after this time) so that:

$$p_{i}\left(t\right)=\{\begin{array}{c} p_{1},\;\;t\leq T\\ p_{2},\;\;t>T \end{array}$$

The binomial log-likelihood of the data given these considerations is:

$$\begin{array}{c} Log\;Likelihood=\\ \sum_{i}\sum_{t}\text{ln}(x_{i}\left(t\right)!)-\ln\left(y_{i}\left(t\right)!\right)-\ln\left(\left(x_{i}\left(t\right)-y_{i}\left(t\right)!\right)\right)+y_{i}\left(t\right).\ln\left(p_{i}\left(t\right)\right)+(x_{i}\left(t\right)-y_{i}\left(t\right)).\ln\left(1-p_{i}\left(t\right)\right) \end{array}$$

This LL sum can now be used as an objective function for our Markov Chain Monte Carlo scheme, to fit our model parameter values so that model outputs match the NNDSS data. However, we find that it can be excessively difficult to explore the parameter space productively (i.e. moving from regions of poorer to relatively better fits to the data) since the binomial likelihood surface can take on high (i.e. better fitting) values in very small (and therefore very hard to locate) regions of parameter space. To attempt to remedy this problem (as well as to account for a more general degree of uncertainty in the disease reporting rate), we introduce into our observation model a further layer of uncertainty such that the proportion of individuals reporting disease is drawn from a Beta distribution, which has two parameters,

$$\pi \left(p_{i}\left(t\right)\right)=Beta\left(\propto \left(t\right),\beta \left(t\right)\right)$$

where the Beta distribution’s parameter values are time-dependent in the same way as we posited above i.e.:

$$\propto \left(t\right),\beta \left(t\right)=\{\begin{array}{l} \propto_{1},\;\beta_{1}\;\;t\leq T\\ \propto_{2},\;\beta_{2}\;\;t>T \end{array}$$

Using this structure for the reporting rate component of the observation model, we can recalculate the likelihood of a particular dataset given the model (removing the subscripts for simplicity of presentation):

$$likehood=\int_{0}^{1}Binomial\left(x,p\right).\pi \left(p\right).dp$$

This integral allows us to compute an average likelihood allowing for the entire distribution of the reporting rate *p*, given its Beta distribution. Because the Beta and Binomial distributions are conjugate, the integral is straightforward.

$$\begin{array}{c} likehood=\frac{\left(\begin{array}{c} x\\ y \end{array}\right)}{B\left(\propto \left(t\right),\beta \left(t\right)\right)}\int_{0}^{1}Binomial\left(x,p\right).\pi \left(p\right).dp\\ =\frac{\left(\begin{array}{c} x\\ y \end{array}\right)}{B\left(\propto ,\beta \right)}\int_{0}^{1}p^{y+\propto -1}.{\left(1-p\right)}^{x-y+\beta -1}.dp \end{array}$$

Where *B*(∝, *β*) is the normalizing constant for the Beta probability distribution. The integrand can be seen to be the probability density function of the Beta distribution *Beta*(∝′, *β*′) with the adjusted (or ‘renormalized’) parameter values, so that:

$$\propto^{\prime }=y+\propto ,\beta^{\prime }=x-y+\beta$$

So the integral above is the normalization constant of the Beta distribution (which is referred to as the Beta function, itself comprised of a set of gamma functions, see below) with the adjusted parameters. The likelihood therefore becomes:

$$\begin{array}{c} likelihood=\frac{\left(\begin{array}{c} x\\ y \end{array}\right).B\left(y+\propto ,x-y+\beta \right)}{B\left(\propto ,\beta \right)}\\ =\frac{\left(\begin{array}{c} x\\ y \end{array}\right).\Gamma \left(y+\propto ).\Gamma (x-y+\beta \right).\Gamma \left(\propto .\beta \right)}{\Gamma \left(\propto ).\Gamma (\beta \right).\Gamma \left(\left(y+\propto ).(x-y+\beta \right)\right)} \end{array}$$

And for all of the data points, the log-likelihood becomes:

$$LL=\sum_{i}\sum_{t}\left[ln\left(\begin{array}{c} x_{i}\left(t\right)\\ y_{i}\left(t\right) \end{array}\right)+\ln\left(\Gamma \left(y_{i}\left(t\right)+\propto \left(t\right))\right)+ln\left(\Gamma (x_{i}\left(t\right)-y_{i}\left(t\right)+\beta \left(t\right)\right)\right)+\ln\left(\Gamma \left(\propto \left(t\right).\beta \left(t\right)\right)\right)-\ln\left(\Gamma \left(\propto \left(t\right))\right)-ln\left(\Gamma (\beta \left(t\right)\right)\right)-\text{ln}\left(\Gamma \left(\left(y_{i}\left(t\right)+\propto \left(t\right)).(x_{i}\left(t\right)-y_{i}\left(t\right)+\beta \left(t\right)\right)\right)\right)\right]$$

## Supporting Information

S1 Text S1 Text includes a brief description of alternative model structures that have been investigated in the recent literature; a table outlining precisely which parameters are allowed to alter and which are fixed for each of the models we investigate; a series of plots outlining the best-fitting model’s fit to the age-dependent incidence of disease cases for each of the years between 1990 and 2009; and a model-based simulation of the case-control study outlined in the main article, showing the gradual effect of an aging cohort of individuals who have been entirely vaccinated with the DTaP rather than the DTP vaccine. (DOCX)
