# Supplementary material for: A Change in Vaccine Efficacy and Duration of Protection Explains Recent Rises in Pertussis Incidence in the United States
Source: PLoS Comput Biol. 2015 Apr 23;11(4):e1004138. doi: 10.1371/journal.pcbi.1004138 (PMC4408109; doi:10.1371/journal.pcbi.1004138)
Supplement: S1 Text — (DOCX) [file pcbi.1004138.s001.docx]

**S1 Text**

*Alternative model structures*


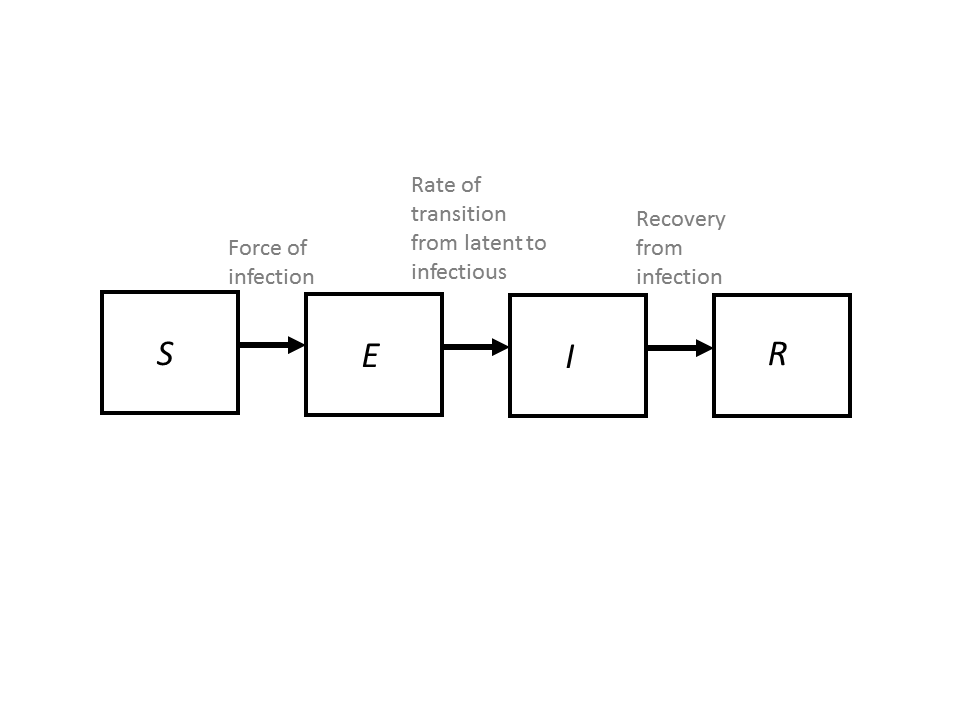


Figure 1: The model of Rohani *et al*[[1](#_ENREF_1)]. This is a simple SEIR model in which a compartment is introduced to represent a latent period during which an individual is infected but not yet infectious. Note also that there is no exit from the recovered compartment, so that once an individual has recovered from an infection (or has been vaccinated) the induced immunity is lifelong.


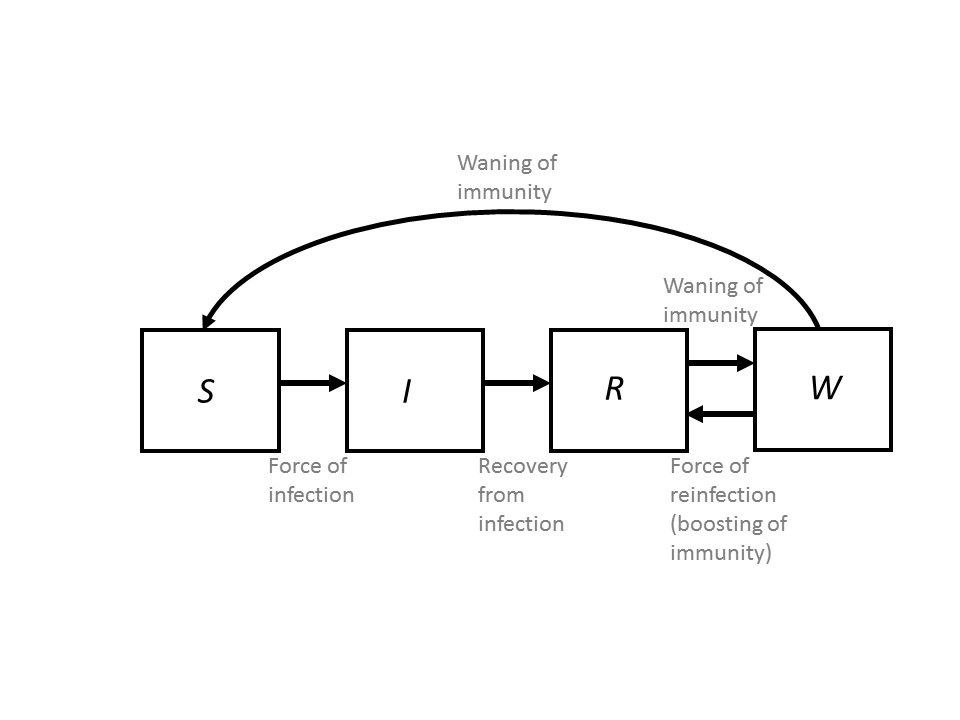


Figure 2: The model of Lavine *et al* [[2](#_ENREF_2)]. This model is conceptually very similar to that of Aguas *et al*[[3](#_ENREF_3)]. The main difference is that individuals who have experienced a prior infection (or vaccination) move into a 'waning' compartment (*W*, above) and, during this period, they may either lose their immunity entirely (and revert to susceptible) or become re-infected and pass back into the recovered (immune) class *R*. Those whose immunity is waning are not infectious in this model. Aside from the minor fact of the precise compartment from which these individuals flow back into the susceptible population, the force of infection also differs between the two models. In the Aguas model, those recovered and immune may still become infected but their susceptibility has decreased. In the Lavine model, the susceptibility to 're-infection' of those in the W class vastly increases: their immune system is primed and they are more likely to find it boosted by very infrequent contact with the pathogen.

*Further details of model comparisons*

In the following table, we outline the specific parameters that are altered independently in our model comparison exercise, as detailed in the main text. The symbols denoting the rate of exit from the naturally infected compartment is given by *α* and that for the whole-cell vaccinated compartment, *γ.* The vaccine efficacy of the whole-cell and acellular vaccines are denoted *VE*_wP_ and *VE*_aP_, and their durations of protection by *dur*_wP_ and *dur*_aP_.

| **Model** | **Description** | **Parameter values** |
| --- | --- | --- |
| **1** | Vaccination equivalent to natural infection; no change in duration or effectiveness | *γ=α*  *VE*_wP_=*VE*_aP_  *dur*_wP_=*dur*_aP_ |
| **2** | Vaccination equivalent to natural infection; no change in duration; change in effectiveness in 1992-1998 | *γ=α*  *VE*_wP_*≠VE*_aP_  *dur*_wP_=*dur*_aP_ |
| **3** | Vaccination equivalent to natural infection; change in duration in 1992-1998; no change in effectiveness | *γ=α*  *VE*_wP_=*VE*_aP_  *dur*_wP_*≠dur*_aP_ |
| **4** | Vaccination duration different from natural infection; no change in duration or effectiveness | *γ≠α*  *VE*_wP_=*VE*_aP_  *dur*_wP_=*dur*_aP_ |
| **5** | Vaccination equivalent to natural infection; change in duration in 1992-1998; change in effectiveness in 1992-1998 | *γ=α*  *VE*_wP_*≠VE*_aP_  *dur*_wP_*≠dur*_aP_ |
| **6** | Vaccination duration different from natural infection; no change in duration; change in effectiveness in 1992-1998 | *γ≠α*  *VE*_wP_*≠VE*_aP_  *dur*_wP_=*dur*_aP_ |
| **7** | Vaccination duration different from natural infection; change in duration in 1992-1998; no change in effectiveness | *γ≠α*  *VE*_wP_*=VE*_aP_  *dur*_wP_*≠dur*_aP_ |
| **8** | Vaccination duration different from natural infection; change in duration in 1992-1998; change in effectiveness in 1992-1998 | *γ≠α*  *VE*_wP_*≠VE*_aP_  *dur*_wP_*≠dur*_aP_ |

*Full annual age-dependent incidence data compared with model generated output*

Below we show the annual age-dependent disease incidence in the US between 1990 and 2009 (black dots) and the model-generated means (blue lines) and the corresponding uncertainty envelopes (grey shaded regions, CIs as for the shaded envelopes in the main text) for the best fitting model (Model 8).


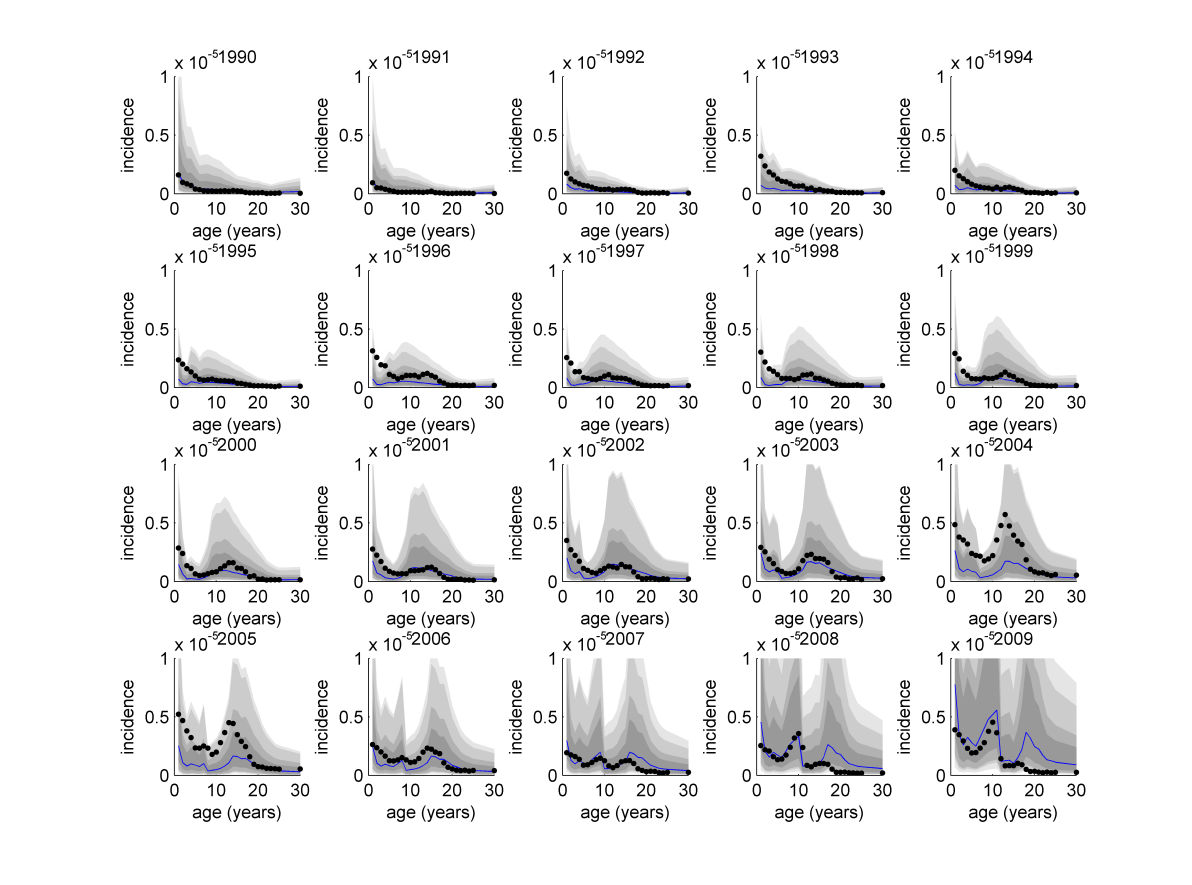


Figure 3: Annual age-dependent (to age 30 years) incidence of pertussis disease cases for the years 1990-2009. Gray shaded envelopes indicate 50%, 90%, 95%, and 99% credible intervals from the model parameter and uncertainty estimation. Black points indicate disease incidence data collected by the NNDSS.

*Further analysis relating to simulated case-control VE studies*

An equivalent simulated case-control study to that outlined in the main text, was also performed for each of the years between 1990 and 2009, using the best-fitting model. The utility of such a simulated study is to examine whether there are any changes in the pattern of measured VE, following the administration of the 5^th^ dose of vaccine, over time. Figure 4 illustrates a sequence VE simulations of the same case-control study, had it been performed in each year 2000-2006. We find that the decline in VE occurs in steps as each annual cohort of children vaccinated entirely with the acellular vaccine enters the simulated study. Within the given years 2003-2005 the VE clearly rises, with time since final dose, due to the specific year at which the entire vaccination schedule was changed from wP to aP in the simulation. Such a clearly-defined VE change is unlikely to be observed in real case data, though such an upturn may be responsible for the slightly higher final data point in Figure 5 from Misegades et al [[4](#_ENREF_4)], for which there may remain a small proportion of individuals with mixed aP and wP or entirely wP vaccination histories.


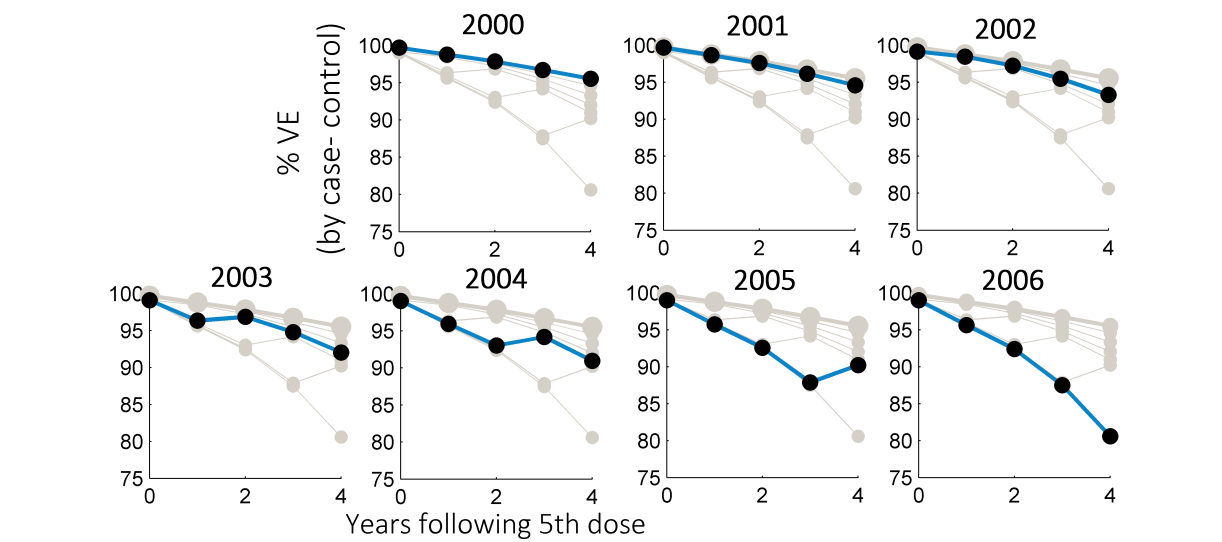


Figure 4: Model-generated VE profiles for the years 2000-2006 following the delivery of the final vaccine dose (black dots joined by blue lines; faint gray lines show the de-emphasised full set of VE curves). These were obtained by the same simulated case-control method used to generate the results shown in Figure 5 (main text).

*Projections of the best-fitting model into the future, following TDaP administration*

In order to guage the possible future impact of increased coverage levels of the adolescent booster vaccine Tdap, we simulated increased coverage levels from zero to 75%. Figure 5 shows the effect of performing such a forward simulation. This figure shows that an oscillatory pattern of incidence over the next 2 decades is a possibility, with ‘epidemic’ years occurring at least once a decade. Increased effectiveness (i.e. here = coverage*efficacy) of the Tdap vaccine could lead to an increase in the interepidemic period as well as an overall decrease of the incidence level.


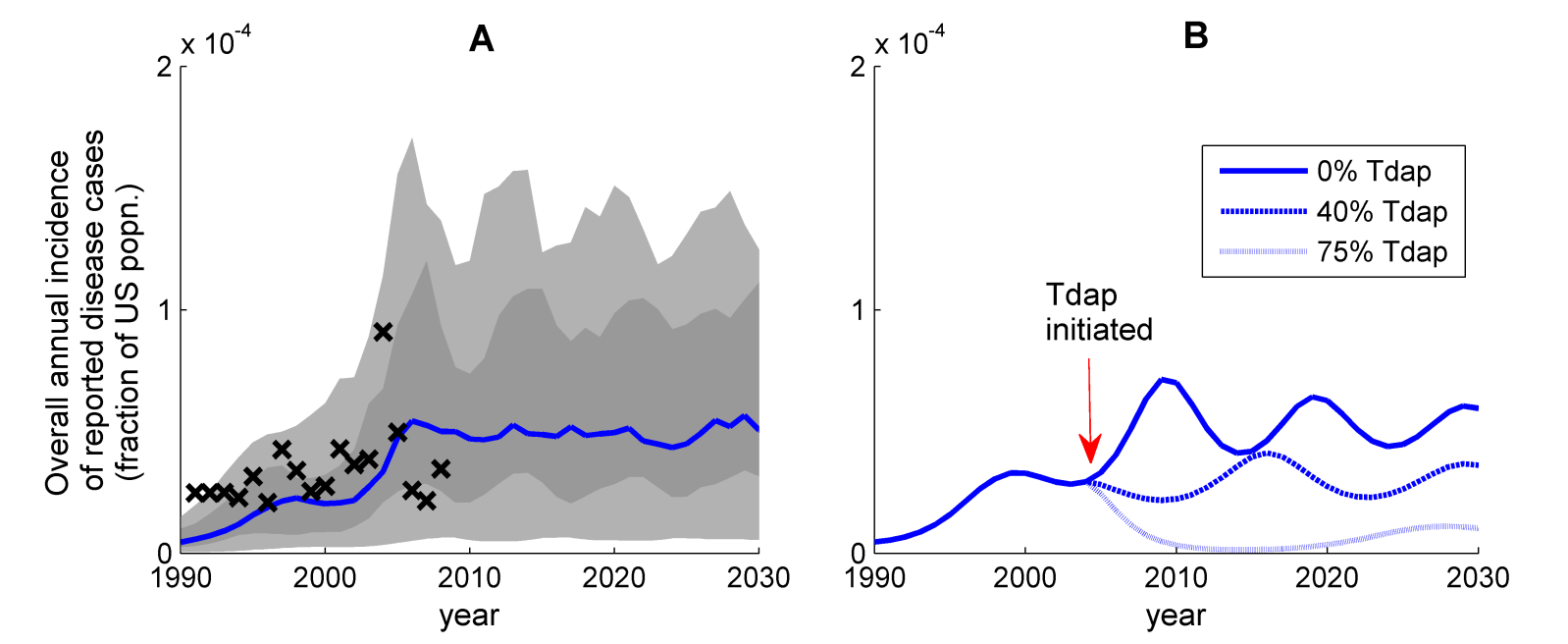


Figure 5: Model-generated forecasts 20 years into the future for the best fitting model in the main text (A). The mean of 1000 simulations, with parameter values drawn from the parameter posterior distributions, is shown by the blue solid line and the 50% and 75% interval envelopes are given by the inner and outer grey shaded regions. (B) shows the effect of administering the Tdap vaccination at increasing levels of effectiveness (effectiveness here = coverage*efficacy) to the adolescent population beginning in 2004, for a single simulation run.

*Parameter posterior distributions*


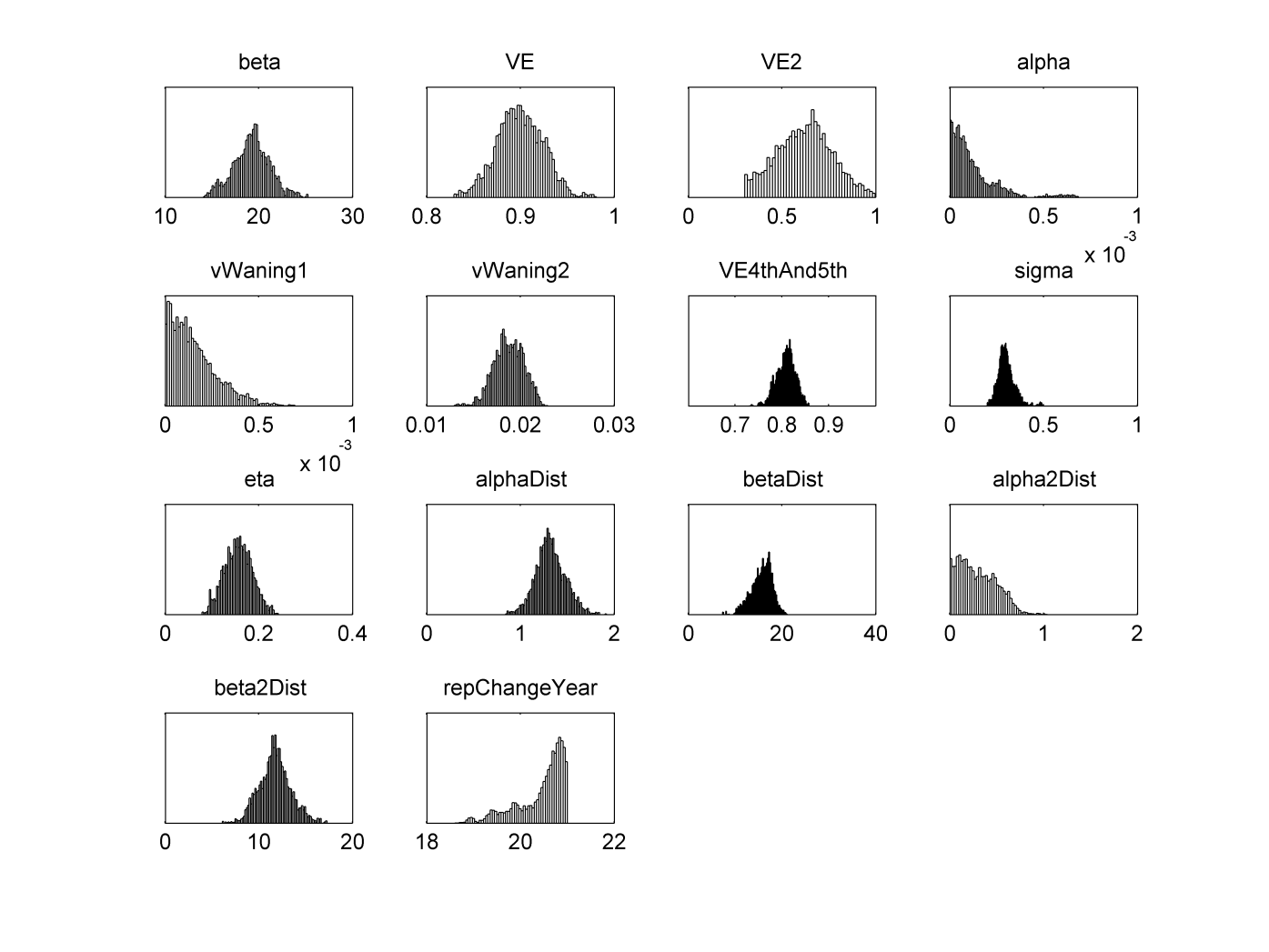


Figure 6: Posterior parameter distributions for the 11 parameters of the best-fitting model detailed in the main text. The parameters are as follows: beta—transmission coefficient; VE—vaccine efficacy of the first 3 doses of the wP vaccine; VE2—unused variable for this analysis (previously aP vaccine efficacy); alpha—rate of decay of immunity from natural infection; vWaning1—rate of decay of immunity from wP vaccine; vWaning2—rate of decay of immunity from aP vaccine; VE4thAnd5th—aP vaccine efficacy; sigma—relative susceptibility of individuals who have been infected to those who are naïve; eta—relative infectiousness of those who have been infected to those who are naïve; alphaDist—Beta distribution ‘alpha’ parameter prior to a change in reporting; betaDist—Beta distribution ‘beta’ parameter prior to a change in reporting; alpha2Dist—Beta distribution ‘alpha’ parameter following a change in reporting; beta2Dist—Beta distribution ‘beta’ parameter following a change in reporting; repChangeYear—year after 1990 in which a reporting change occurs.

**References**

1. Rohani P, Zhong X, King AA (2010) Contact network structure explains the changing epidemiology of pertussis. Science 330: 982-985.

2. Lavine JS, King AA, Bjornstad ON (2011) Natural immune boosting in pertussis dynamics and the potential for long-term vaccine failure. Proc Natl Acad Sci U S A 108: 7259-7264.

3. Aguas R, Goncalves G, Gomes MG (2006) Pertussis: increasing disease as a consequence of reducing transmission. Lancet Infect Dis 6: 112-117.

4. Misegades LK, Winter K, Harriman K, Talarico J, Messonnier NE, et al. (2012) Association of childhood pertussis with receipt of 5 doses of pertussis vaccine by time since last vaccine dose, California, 2010. JAMA 308: 2126-2132.
